# The Preparation and Optical Properties of Novel LiLa(MoO_4_)_2_:Sm^3+^,Eu^3+^ Red Phosphor

**DOI:** 10.3390/ma11020297

**Published:** 2018-02-14

**Authors:** Jiaxi Wang, Li Luo, Baoyu Huang, Jingqi He, Wei Zhang, Weiren Zhao, Jianqing Wang

**Affiliations:** 1 School of Physics & Optoelectric Engineering, Guangdong University of Technology, Guangzhou Higher Education Mega Centre, Guangzhou 510006, China; 18635208773@163.com (J.W.); 13503036067@163.com (B.H.); 13690103732@163.com (J.H.); weizh55@gdut.edu.cn (W.Z.); zwren@gdut.edu.cn (W.Z.); 2 Guangzhou LEDteen Optoelectronics Co., Ltd. 2F A4 Building, No. 11 Kaiyuan Avenue, Science City, Guangzhou Hi-tech Industrial Development Zone, Guangzhou 510663, China; wangjianqing@ledteen.com

**Keywords:** molybdate, red phosphor, energy transfer, thermal stability

## Abstract

Novel LiLa_1−*x*−*y*_(MoO_4_)_2_:*x*Sm^3+^,*y*Eu^3+^ (in short: LL_1−*x*−*y*_M:*x*Sm^3+^,*y*Eu^3+^) double molybdate red phosphors were synthesized by a solid-state reaction at as low temperature as 610 °C. The optimal doping concentration of Sm^3+^ in LiLa_1−*x*_(MoO_4_)_2_:*x*Sm^3+^ (LL_1−*x*_M:*x*Sm^3+^) phosphor is *x = *0.05 and higher concentrations lead to emission quenching by the electric dipole—electric dipole mechanism. In the samples co-doped with Eu^3+^ ions, the absorption spectrum in the near ultraviolet and blue regions became broader and stronger than these of the Sm^3+^ single-doped samples. The efficient energy transfer from Sm^3+^ to Eu^3+^ was found and the energy transfer efficiency was calculated. Under the excitation at 403 nm, the chromaticity coordinates of LL_0.95−*y*_M:0.05Sm^3+^,*y*Eu^3+^ approach to the NTSC standard values (0.670, 0.330) continuously with increasing Eu^3+^ doping concentration. The phosphor exhibits high luminous efficiency under near UV or blue light excitation and remarkable thermal stability. At 150 °C, the integrated emission intensity of the Eu^3+^ remained 85% of the initial intensity at room temperature and the activation energy is calculated to be 0.254 eV. The addition of the LL_0.83_M:0.05Sm^3+^,0.12Eu^3+^ red phosphors can improve the color purity and reduce the correlated color temperature of WLED lamps. Hence, LL_1−*x*−*y*_M:*x*Sm^3+^,*y*Eu^3+^ is a promising WLED red phosphor.

## 1. Introduction

With the problem of environmental pollution and energy crisis becoming increasingly prominent in recent years, the white light emitting diode (WLED) has attracted wide attention due to the advantages of energy saving, long lifetime, high efficiency and environmental friendliness [1,2]. These attributes have made WLEDs to be the perfect candidate for the next generation of light sources. WLEDs can be used widely in lighting, backlighting, display, indicating areas, urban lighting, etc. [3]. White light can be produced by exciting multi-phosphors by a UV-LED, or by the combination of a blue LED chip with a yellow phosphor, or by blending multi-LEDs [4]. The combination of the commercial YAG:Ce^3+^ yellow phosphor pumped by a blue InGaN chip has some drawbacks which are difficult to overcome: a high correlated color temperature (CCT) and low color rendering index (CRI) due to lack of red component. Hence this color cannot meet indoor light requirements which require warm white light and an excellent CRI [5]. In addition, the blue chip InGaN and yellow phosphor possess a different failure rate and in the case of long working hours this may cause chromatic aberration and reduced white performance [6,7,8,9,10]. To solve this problem, researchers have developed red, green and blue emission phosphors using near ultraviolet (NUV: 350~410 nm) excitation [11,12]. When combined with a GaN/InGaN chip, light of higher CRI, better lighting uniformity and high luminous efficiency can be achieved. Currently, WLED phosphors have made great progress: especially for blue and green phosphors. However, research and development of red phosphors is required [13], since the current commonly used Eu^2+^ and Mn^4+^-based red emitting phosphors (such as Sr_2_Si_5_N_8_:Eu^2+^ [14], CaAlSiN_3_:Eu^2+^ [15], Sr[LiAl_3_N_4_]:Eu^2+^ [16,17], K_2_SiF_6_:Mn^4+^ [18,19]) for high performance WLED have some problems such as a wide band emission, unsatisfactory color quality and a relative high production cost, or low stability and easily generating poisonous gas, thereby restricting the development of WLEDs. Therefore, the development of narrow-bandwidth red phosphors which can be synthesized at a moderate temperature and in an ambient atmosphere and with stable and efficient luminescent properties has important significance [20,21].

In recent years, the luminescence properties of Sm^3+^ and Eu^3+^ rare earth ions have attracted much attention because they have potential applications as WLED red phosphors [22,23,24,25,26]. These phosphors work under the sharp 4f—4f absorption excitations of Sm^3+^/Eu^3+^ at around 400 nm but unfortunately these excitations individually show weak intensities because the absorption transitions are forbidden by the parity selection rule to first order. Besides, their full widths at half maximum are narrow and not tolerant to the emission wavelength shifts of UV LED chips [11]. The Sm^3+^ or Eu^3+^-doped phosphor cannot be efficiently excited by a near ultraviolet or a blue LED chip alone, which limits the Sm^3+^ or Eu^3+^ single-doped phosphor application in WLED. To solve this problem, many researchers have prepared Eu^3+^-Sm^3+^ co-doped phosphors [27,28,29,30,31,32,33,34,35,36,37,38,39,40,41], aimed at improvement of the absorption in the near-ultraviolet region around 400 nm or the blue region around 460 nm to improve emission performance.

Double molybdates ALn(MoO_4_)_2_ (where A is an alkali metal ion and Ln is a trivalent rare earth ion) with the scheelite structure are well known for their laser applications [42,43,44,45,46] and are considered to be promising luminescent host candidates due to their excellent optical properties and physico-chemical stability [11]. In these materials, the central Mo^6+^ metal cation is surrounded by four oxygen neighbors in a tetragonal shape and it is isolated from other Mo^6+^ cations. The Li^+^/La^3+^ cations are surrounded by eight oxygen atoms from different MoO_4_ tetrahedra. The random distribution of Li^+^ and La^3+^ produces a low symmetry environment. Hence, the inhomogeneous broadening of the bands occurs when rare earth ions are doped to replace La^3+^ ions in these crystals [47]. Many groups have made efforts to develop such phosphors, for example: LiLa(MoO_4_)_2_:Eu^3+^ [24], LiY(MoO_4_)_2_:Sm^3+^ [23], KY_1−*x*_Ln*_x_*(MoO_4_)_2_:Ln (Ln = Sm^3+^,Eu^3+^) [11], NaGd(MoO_4_)_2_:Sm^3+^,Eu^3+^ [48], etc. The energy transfer from Sm^3+^ to Eu^3+^ has been found and the transfer mechanism and efficiency were studied. However, to the best of our knowledge, no research on LiLa(MoO_4_)_2_:Sm^3+^,Eu^3+^-based phosphors with orthorhombic structure has been reported on.

Based on the above survey, to find an efficient WLED red phosphor, Sm^3+^ and Eu^3+^ co-doped LiLa(MoO_4_)_2_ phosphors were successfully synthesized at 610 °C via the conventional solid-state method. The Sm^3+^ single-doped LiLa(MoO_4_)_2_ phosphors exhibited orange-red emission and the Eu^3+^ co-doping improved light color purity. The synthesis temperature of the molybdate matrix is lower than previously reported value [24,25,27,28,29,30,31,32,33,34,35,36]. The low synthesis temperature was reached employing nano-scale raw materials and longtime grinding of the reactants. Moreover, previous researches on Sm^3+^ and Eu^3+^ codoped phosphors were mainly focused on the synthesis and red light emitting properties. However, such important properties as thermal stability and luminous efficiency were not reported. In particular, a blue chip excited WLED+phosphor structure was never evaluated. The main objective of this work is to carry out a detailed study on the energy transfer, color adjustment, thermal stability, and LED application oriented performance of LiLa(MoO_4_)_2_:Sm^3+^,Eu^3+^ red phosphor. The luminous efficiency and thermal stability of our sample are studied and compared with commercial Y_2_O_3_:Eu^3+^ red phosphor. In addition, an WLED lamp possessing high luminous efficacy and low CCT has been fabricated using our sample and the optical properties have been investigated.

## 2. Experimental

### 2.1. Sample Preparation

A series of LiLa(MoO_4_)_2_:Sm^3+^,Eu^3+^ phosphors with different concentrations was prepared by a conventional solid-state reaction method in the air. The chemical reagents, Li_2_CO_3_ (>97%, Guangzhou Chemical Factory, Guangzhou, China), MoO_3_ (>99.5%, Tianjin Zhiyuan Chemicals, Tianjin, China), and La_2_O_3_ (99.99%), Eu_2_O_3_ (99.9%), Sm_2_O_3_ (99.99%) (all from Shanghai Aladdin, Shanghai, China) were used as starting materials. These starting materials were ground in an agate mortar for 1 h with a small amount of ethanol (to help grinding) to form a homogeneous fine powder mixture. Then, the powder mixtures were transferred into corundum crucibles and were pre-fired at 610 °C in an air atmosphere for 6 h. Finally, the samples were gently ground two times to obtain the phosphor powders. The obtained samples were as follows: LiLa_1−*x*_(MoO_4_)_2_:*x*Sm^3+^ (*x *= 0, 0.01, 0.03, 0.05, 0.08, 0.12), henceforth LL_1−*x*_M:*x*Sm^3+^; LiLa_0.95−*y*_(MoO_4_)_2_:0.05Sm^3+^,*y*Eu^3+^ (*y *= 0.01, 0.03, 0.05, 0.8, 0.12), henceforth LL_0.95−*y*_M:0.05Sm^3+^,*y*Eu^3+^; and LiLa_0.95_(MoO_4_)_2_:0.05Eu^3+^, henceforth LL_0.95_M:0.05Eu^3+^.

### 2.2. Characterization of Samples

The phase purity of the prepared phosphors was analyzed by X-ray powder diffraction using Cu Kα radiation (wavelength = 0.15406 nm) at 36 kV tube voltage and 20 mA tube current. The cell parameters were calculated by MDI Jade 6.5 software. The diffuse reflectance spectrum of sample was measured on a Cary 5000 UV-Vis-NIR spectrophotometer attached to an integrating sphere, using polytetrafluoroethene as a standard for measurement. The photoluminescence emission (PL) and photoluminescence excitation (PLE) spectra of the obtained powders were recorded by a Hitachi F-7000 Fluorescence Spectrophotometer (Hitachi, Tokyo, Japan) equipped with a 450 W xenon lamp as excitation source. Lifetime measurements were carried out with an Edinburgh Instruments spectrometer (model FLS 980). Temperature-dependent PL properties were investigated by a Renishaw Spectrometer (Renishaw, Hong Kong, China) equipped with a 325 nm laser and temperature control system.

An WLED lamp was fabricated using a 455 nm blue LED chip pre-coated with LL_0.83_M:0.05Sm^3+^, 0.12Eu^3+^ red phosphor and commercial yellow phosphor encapsulated in a transparent epoxy resin based on the standard LED technology in Guangzhou LEDteen Optoelectronics Co., Ltd. (Guangzhou, China).The pumping LED source is an 455 nm InGaN LED COB (chip on board) in which 12 chips are connected in series; the driving voltage of single chip is 2.9–3.3 V. The commercial yellow phosphor is from Jiangsu Bree Optronics Co., Ltd. (Nanjing, China). The material is rare earth aluminates, the excitation wavelength is 440–455 nm, the peak wavelength is 537 nm, CIE is x=0.380±0.005,y=0.571±0.005, peak half-width bandwidth is 105.7 nm, color purity is 88%, and particle size is 12.5±0.5 μm. The dispensing type silicon encapsulant for LED is ENA6550 A/B (Tianbao Science&Technology Co., Ltd., Taiwan).

## 3. Results and Discussion

### 3.1. X-Ray Diffraction Patterns

**Figure 1:**
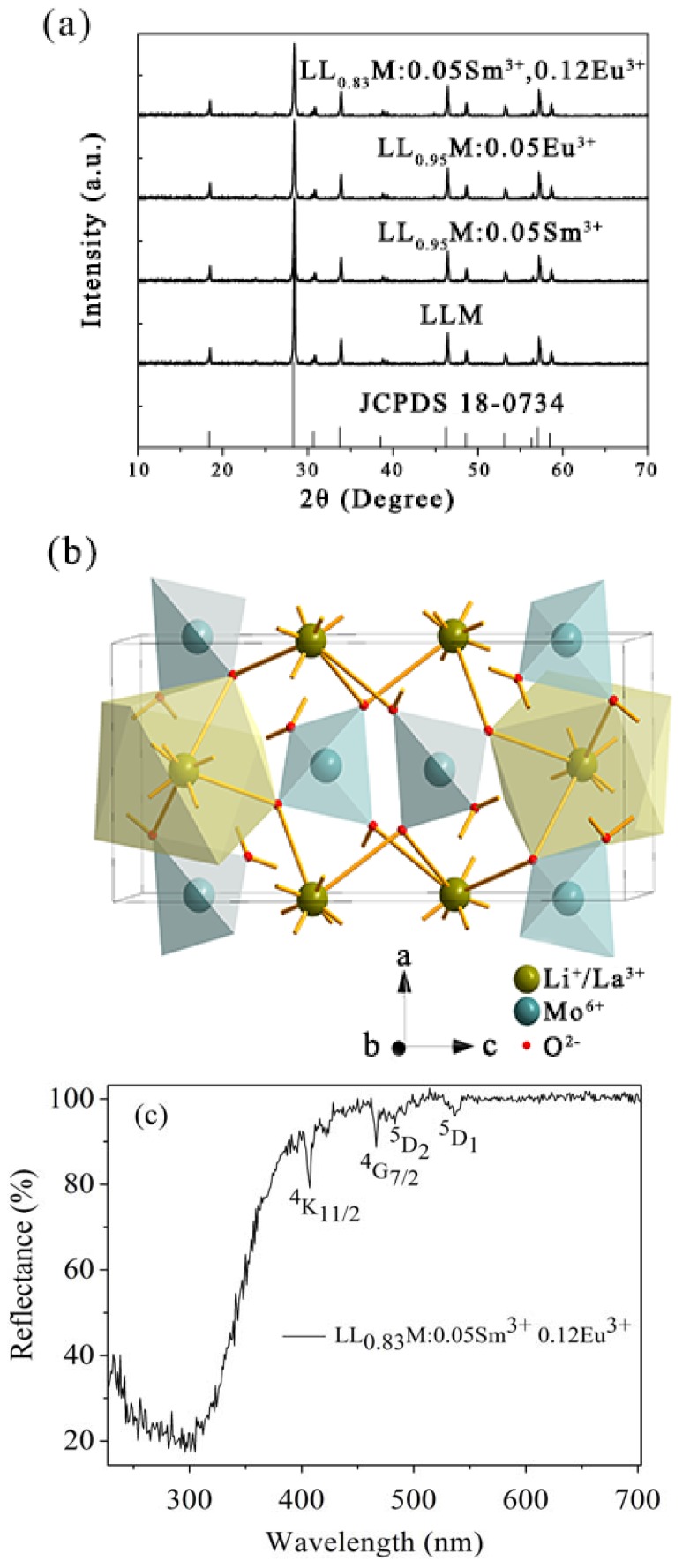
(**a**) The XRD (X-ray powder diffraction) patterns of LiLa(MoO_4_)_2_, LiL_0.95_(MoO_4_)_2_:0.05Sm^3+^, LiLa_0.95_(MoO_4_)_2_:0.05Eu^3+^, LiLa_0.83_(MoO_4_)_2_:0.05Sm^3+^,0.12Eu^3+^ samples with the standard data of LiLa(MoO_4_)_2_ (JCPDS No. 18-0734); (**b**) crystal structure of tetragonal phase LiLa(MoO_4_)_2_; (**c**) The diffuse reflectance spectrum of LiLa_0.83_(MoO_4_)_2_:0.05Sm^3+^,0.12Eu^3+^. Some locations of terminal *J*-multiplets of Sm^3+^ and Eu^3+^ transitions are marked. The electronic ground states are ^6^H_5/2_ (Sm^3+^) and ^7^F_0_ (Eu^3+^).

X-ray powder diffraction is an important method to estimate the phase purity. The X-ray diffractograms of four kinds of samples are shown in Figure 1a. The positions of all diffraction peaks are very close to that of LiLa(MoO_4_)_2_ (LLM) standard card (JCPDS 18-0734) [24]. Thus, the doping with rare earth ions did not change the crystal structure and the samples prepared by this method are of pure LLM phase. According to Bragg’s law, 2dsinθ=nλ, the unit cell group parameters for LLM are calculated as a = b = 5.293 Å, c = 11.598 Å, *Z* = 2, *V* = 324.97 Å^3^ (space group *I*4_1_/a).

Figure 1b shows the LLM tetragonal phase crystal structure diagram drawn by Diamond software. Each Mo^6+^ cation is coordinated by four O^2−^ with formations of MoO_4_ tetrahedrons; Li^+^ and La^3+^ ions are randomly distributed in the MO_8_ polyhedra contacting with MoO_4_ by corners.

Figure 1c displays the diffuse reflectance spectrum of the phosphor. The sample has a broad absorption band in the range of 200–380 nm due to host absorption and obvious absorptions in the longer wavelength region due to the 4f^N^—4f^N^ transitions of Sm^3+^ and Eu^3+^ ions, as indicated.

### 3.2. The Excitation and Emission Spectra of LiLa_1−__x_(MoO_4_)_2_ Doped with Sm^3+^

**Figure 2:**
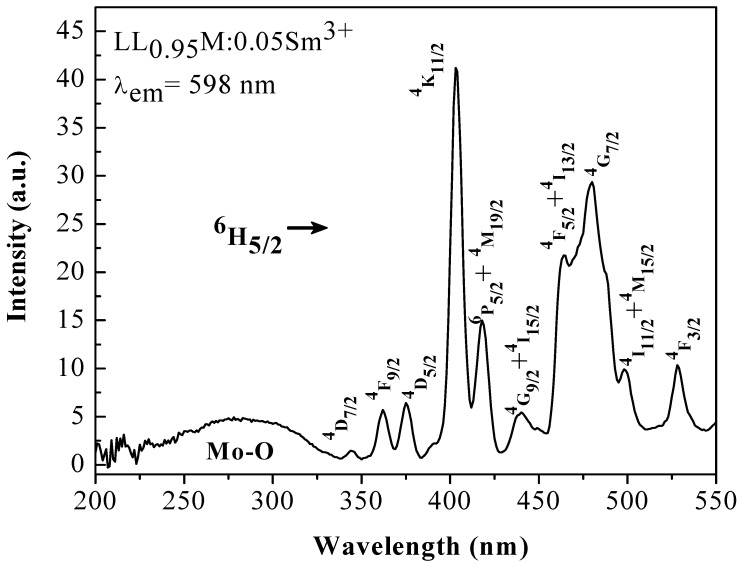
Excitation spectrum of LL_0.95_M:0.05Sm^3+^ phosphor, monitoring the emission at 598 nm.

The electronic ground state of Sm^3+^ is ^6^H_5/2_. The excitation spectrum of LL_0.95_M:0.05Sm^3+^ recorded by monitoring the ^4^G_5/2_ → ^6^H_5/2_ emission of Sm^3+^ at 598 nm is shown in Figure 2. The excitation spectra are composed of a broad band in the ultraviolet region and a number of narrow-band peaks in the near-ultraviolet to visible region. There is a wide absorption band from 230 to 320 nm, attributed to the charge transfer transition O^2−^ → Mo^6+^ due to the transition of a 2*p* electron of O^2−^ to the *d* orbital of Mo^6+^ [48]. The narrow bands detected over 340–550 nm are the typical weak, sharp 4f—4f transitions of rare earth ions and the terminal states of Sm^3+^ are labeled. The appearance of the charge transfer band is beneficial to the absorption of UV photons. The energy absorbed by the host is then transferred to the rare earth ion. Also, the absorption band of ^6^H_5/2_ → ^4^K_11/2_ at 403 nm is the strongest of the Sm^3+^ 4f—4f transitions and its wavelength is well matched with that of near UV LED chips.

**Figure 3:**
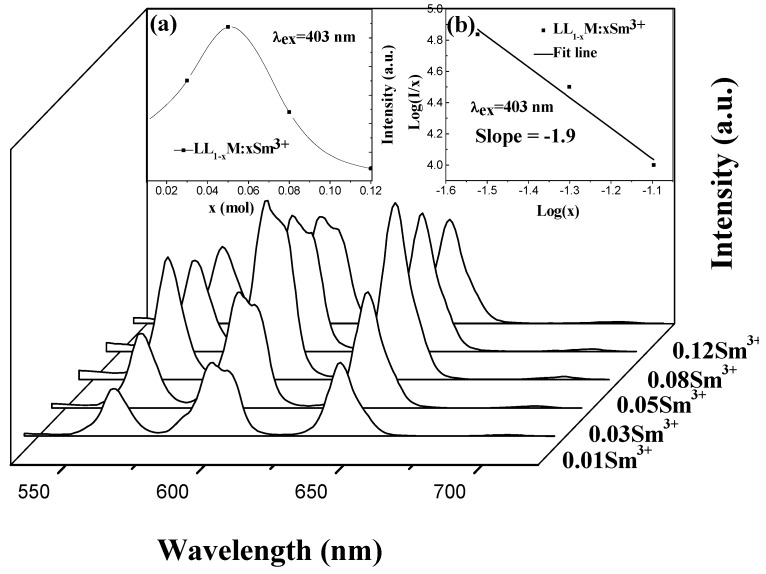
The emission spectra of LL_1−*x*_M:*x*Sm^3+^ (*x *= 0.01, 0.03, 0.05, 0.08, 0.12) under 403 nm excitation. The inset (**a**) shows the relative luminescence intensity at 598 nm as a function of Sm^3+^ concentration; and (**b**) shows the relationship of log(*I*/*x*) vs. log(*x*) for the LL_1−*x*_M:*x*Sm^3+^ phosphors.

A series of LL_1−*x*_M:*x*Sm^3+^ phosphors with different x was prepared to study the relationship between luminescence intensity and the doping concentration of Sm^3+^. Figure 3 shows the relevant emission spectra under 403 nm excitation. The LL_1−*x*_M:*x*Sm^3+^ samples have three emission peaks at 562 nm (^4^G_5/2_ → ^6^H_5/2_), 598 nm (^4^G_5/2_ → ^6^H_7/2_) and 644 nm (^4^G_5/2_ → ^6^H_9/2_) [49,50]. The band at 598 nm corresponds to yellow-orange light and that at 644 nm to deep red, so LL_1−*x*_M:*x*Sm^3+^ samples emit red-orange light, having potential applications in the LCD back light and white LED. According to magnetic dipole and electric dipole transition selection rules, when Sm^3+^ is at the inversion center of the host crystal, the emission of Sm^3+^ is predominantly from the ^4^G_5/2_ → ^6^H_7/2_ (598 nm) magnetic dipole allowed transition. When Sm^3+^ ion is not at a site with inversion symmetry, the emission of Sm^3+^ is predominantly from the ^4^G_5/2_ → ^6^H_9/2_ (644 nm) forced electric dipole transition, but the ^4^G_5/2_ → ^6^H_7/2_ transition also gains forced electric dipole character. In the present case, the emission intensity at 598 nm is similar to that at 644 nm.

With the Sm^3+^ ion concentration increase, the emission intensity increases and then decreases after a maximum value. When the Sm^3+^ doping content is *x* = 0.05 in LL_1−*x*_M:*x*Sm^3+^, the emission intensity reaches the maximum value. The concentration quenching phenomenon is depicted in the inset Figure 3a.

According to the theory of Dexter, the mechanism of photoluminescence concentration quenching in inorganic non-conductive materials is the electric multipole interaction between the ions [51]. When the activator concentration, X, is large enough, there is relationship between the emission intensity *I* and the activator concentration *x*: *I*/*x* α [β(*x*)^θ/3^]^−1^ or log(*I*/*x*) = *c *− (θ/3)log*x* [52], where β is constant, and θ = 6, 8 or 10 corresponds to electric dipole-electric dipole, electric dipole-electric quadrupole or electric quadrupole-electric quadrupole interaction, respectively [52]. The emission spectra of LL_1−*x*_M:*x*Sm^3+^ were measured under 403 nm excitation and the curve lg(I/x) vs. lgx was fitted, as it is shown in Figure 3b. The slope of the straight line is −θ/3 = −1.9, so that θ = 5.7~6. Hence, the concentration quenching mechanism in LL_1−*x*_M:*x*Sm^3+^ is by electric dipole—electric dipole interaction. This concentration quenching mechanism is consistent with that in NaGd(MoO_4_)_2_:Sm^3+^,Eu^3+^ [48].

### 3.3. Energy Transfer from Sm^3+^ to Eu^3+^ in LiLa_1−__x_(MoO_4_)_2_ Doped with Eu^3+^ and Sm^3+^

**Figure 4:**
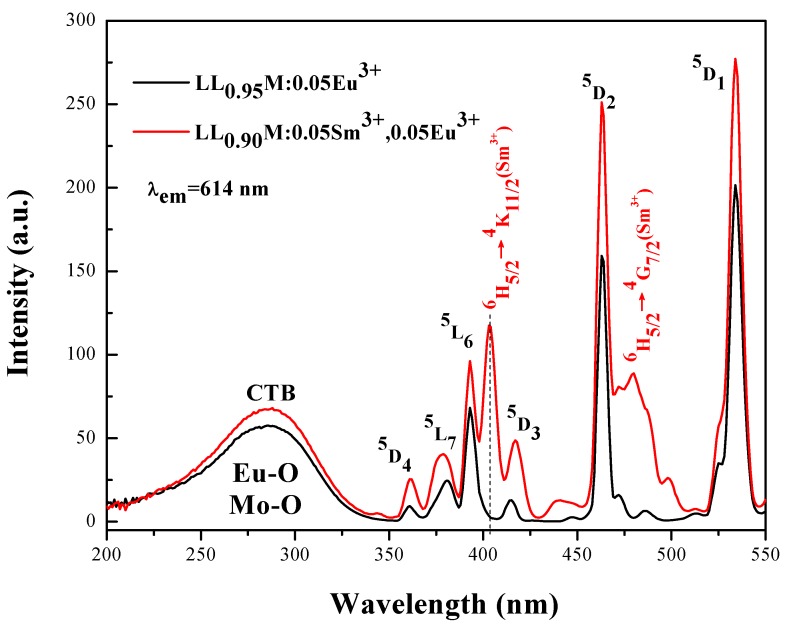
The PLE (photoluminescence excitation) spectrum of LL_0.95_M:0.05Eu^3+^ and LL_0.90_M:0.05Sm^3+^,0.05Eu^3+^ phosphors by monitoring the ^5^D_0_ → ^7^F_2_ emission of Eu^3+^ at 614 nm.

In Figure 4 is shown the excitation spectra of LL_0.95_M:0.05Eu^3+^ and LL_0.90_M:0.05Sm^3+^,0.05Eu^3+^ phosphors by monitoring the ^5^D_0_ → ^7^F_2_ emission of Eu^3+^ at 614 nm. LL_0.95_M:0.05Eu^3+^ has a wide charge transfer band in the UV region between 200 nm and 350 nm, peaking at 287 nm. In addition to the charge transfer band, there are characteristic excitation peaks of the Eu^3+^ at 360 nm (^7^F_0_ → ^5^D_4_), 381 nm (^7^F_0_ → ^5^L_7_), 393 nm (^7^F_0_ → ^5^L_6_), 415 nm (^7^F_0_ → ^5^D_3_), 463 nm (^7^F_0_ → ^5^D_2_) and 534 nm (^7^F_1_ → ^5^D_1_). This indicates that Eu^3+^ in the LLM matrix has a wide absorption range, beneficial to improving the efficiency of the sample in the application of LED. Also, in Figure 4, the excitation spectrum of the co-doped sample LLM:0.05Eu^3+^,0.05Sm^3+^ are shown, as monitored by the ^5^D_0_ → ^7^F_2_ emission of Eu^3+^ at 614 nm. Two new absorption peaks appear after co-doping with Sm^3+^, located at 403 nm (^6^H_5/2_ → ^4^K_11/2_) and 460 nm (^6^H_5/2_ → ^4^G_7/2_), thereby broadening the excitation spectrum and indicating energy transfer from Sm^3+^ to Eu^3+^ ion in the LLM crystal. As seen in Figure 4, the absorption intensity at 403 nm is larger than that at 393 nm, so we can use 403 nm as the excitation wavelength to study the optical properties of the co-doped sample. In addition, all of the excitation peaks are enhanced in the co-doped sample, compared with the Eu^3+^-doped sample. Hence, Sm^3+^ also acts as a sensitizer, which can effectively absorb the excitation energy and transfer it to the activator, so that the luminous efficiency of the material can be enhanced. The excitation spectrum of the co-doped powder matches more closely with the emission wavelength of the LED chip.

**Figure 5:**
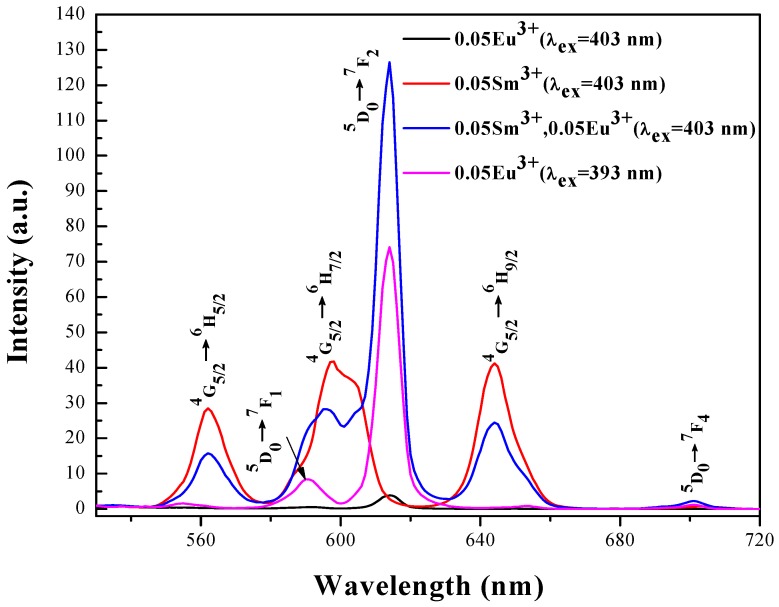
Emission spectra of LL_0.95_M:0.05Eu^3+^, LL_0.95_M:0.05Sm^3+^ and LL_0.90_M:0.05Sm^3+^, 0.05Eu^3+^ (λ_ex_ = 403 nm) compared with the emission spectra of LL_0.95_M:0.05Eu^3+^ (λ_ex_ = 393 nm).

LL_0.95_M:0.05Eu^3+^ has three emission bands at ~590, 614 and 701 nm under the excitation of 393 nm, corresponding to the ^5^D_0_ → ^7^F_1_, ^7^F_2_ and ^7^F_4_ transitions, as shown in Figure 5. The emission intensity at 614 nm is much larger than at 590 nm, which indicates that Eu^3+^ was located in an asymmetric crystal field [53,54]. As it can be reasonably assumed, the Eu^3+^ ion (r = 0.107 nm) replaces La^3+^ (r = 0.106 nm) instead of Li^+^ (r = 0.076 nm) in the crystal because of the same valence and similar radius. When the excitation wavelength was changed to 403 nm, the emission spectrum of LL_0.95_M:0.05Eu^3+^ showed a very weak peak at 614 nm. This indicates that LL_0.95_M:0.05Eu^3+^ cannot be efficiently excited by the 403 nm radiation. As shown in Figure 5, the emission spectrum of LL_0.95_M:0.05Sm^3+^ under 403 nm excitation and it demonstrates that the emission spectrum of LL_0.95_M:0.05Sm^3+^,0.05Eu^3+^ is a superposition of emission spectra of the Sm^3+^ and Eu^3+^ ions. After co-doping Sm^3+^ and Eu^3+^ ions, the characteristic peaks of Sm^3+^ are reduced in intensity from the singly doped LL_0.95_M:0.05Sm^3+^, whereas the characteristic peaks of Eu^3+ ^are stronger than in LL_0.95_M:0.05Eu^3+^ excited by 393 nm radiation. This also confirms the existence of the energy transfer from Sm^3+^ to Eu^3+^. The LL_0.95_M:0.05Sm^3+^ phosphor has the advantage of low synthesis temperature, simple preparation method and high luminous efficiency but it needs for some improvement to be used in practical applications: such as its color purity, which is far from the standard red region and its near UV/blue light absorption is not strong enough. These problems can be solved by the incorporation of Eu^3+^.

**Figure 6:**
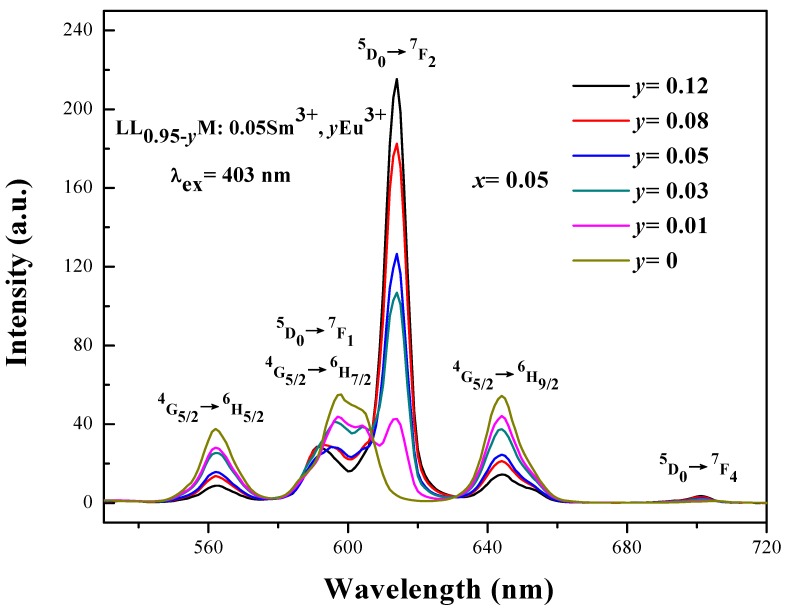
The PL (photoluminescence emission) spectrum of LL_0.95−*y*_M:0.05Sm^3+^,*y*Eu^3+^ (*y* = 0, 0.01, 0.03, 0.05, 0.08, 0.12) under the excitation of 403 nm.

The emission spectra of LL_0.95−*y*_M:0.05Sm^3+^,*y*Eu^3+^ (*y* = 0, 0.01, 0.03, 0.05, 0.08, 0.12) under 403 nm excitation are shown in Figure 6. With the Eu^3+^ concentration increase, the luminescence intensity of Sm^3+^ gradually decreased while the ^5^D_0_ → ^7^F_2_ (614 nm) emission of Eu^3+^ is continuously enhanced.

**Figure 7:**
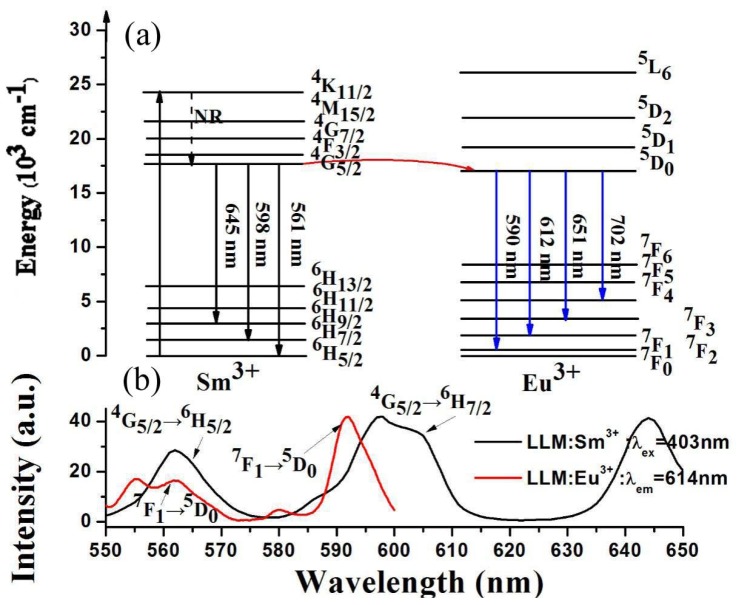
(**a**) The schematic level diagram showing the energy transfer process from Sm^3+^ → Eu^3+^; (**b**) The spectrum overlap between the emission of Sm^3+^ and the excitation of Eu^3+^.

The schematic energy level diagram depicted in Figure 7a indicates that the energy transfer pathway from Sm^3+^ → Eu^3+^ is probably from ^4^G_5/2_ level of Sm^3+^ to ^5^D_0_ level of Eu^3+^, which is confirmed by the spectrum overlap between the emission of Sm^3+^ and the excitation of Eu^3+^, as shown in Figure 7b.

**Figure 8:**
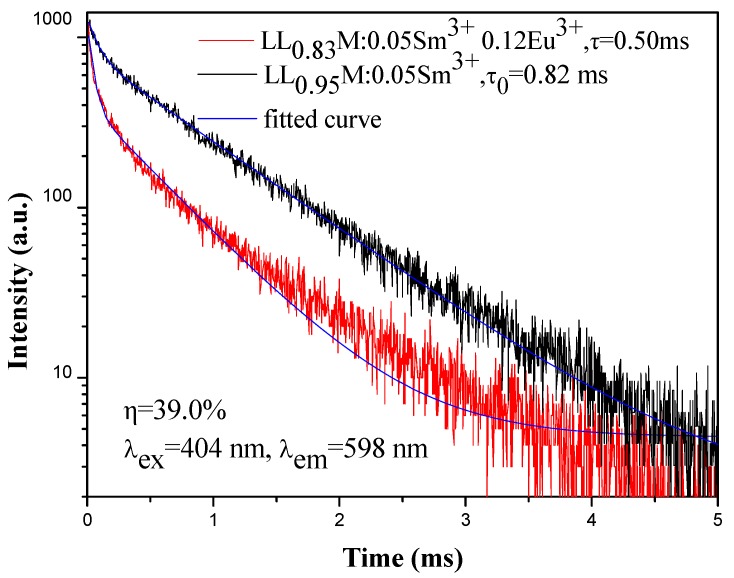
Decay curves of ^4^G_5/2_ Sm^3+^ luminescence of LLM:0.05Sm^3+^ and LLM:0.05Sm^3+^,0.12Eu^3+^, under the excitation of 403 nm and monitored at 598 nm. The luminescent lifetimes (*τ*) and the energy transfer efficiency from Sm^3+^ to Eu^3+^ are indicated.

In order to better understand the energy transfer processes in the LLM:Sm^3+^,Eu^3+^ phosphors, the luminescent dynamics for the ^4^G_5/2_ → ^6^H_7/2_ transition of Sm^3+^ ions at 598 nm was studied. The PL decay curves of Sm^3+^ emission from LLM:Sm^3+^,Eu^3+^ phosphors excited at 403 nm and monitored at 598 nm are shown in Figure 8 and they are fitted by the following double exponential function:

(1) $$I(t)=I_{0}+A_{1}\exp(-t/\tau_{1})+A_{2}\exp(-t/\tau_{2})$$

where *I*(*t*) and *I*_0_ are the luminescence intensities at time *t* and ∞, respectively. *A*_1_ and *A*_2_ are fitting parameters. *τ*_1_, *τ*_2_ are the fast decay and slow decay lifetimes, respectively. The average decay time was calculated using the following formula

(2) $$\tau =(A_{1}\tau_{1}^{2}+A_{2}\tau_{2}^{2})/(A_{1}\tau_{1}^{}+A_{2}\tau_{2}^{})$$

When co-doping with Eu^3+^, the Sm^3+ 4^G_5/2_ lifetime decreases due to energy transfer from Sm^3+^ to Eu^3+^. The energy transfer efficiency (*η_ET_*) from the sensitizer Sm^3+^ to the activator Eu^3+^ can be calculated by the following formula [55]:

(3) $$\eta_{ET}=1-\frac{\tau }{\tau_{0}}$$

where *τ*_0_ is the average decay lifetime of the sensitizer (Sm^3+^) and *τ* is the average decay lifetime of the sensitizer in the presence of the activator (Eu^3+^). The energy transfer efficiency from Sm^3+^ to Eu^3+^ is calculated to be 39.0%.

### 3.4. Color Coordinates of LL_0.95−__y_M:0.05Sm^3+^,yEu^3+^ Phosphors

**Figure 9:**
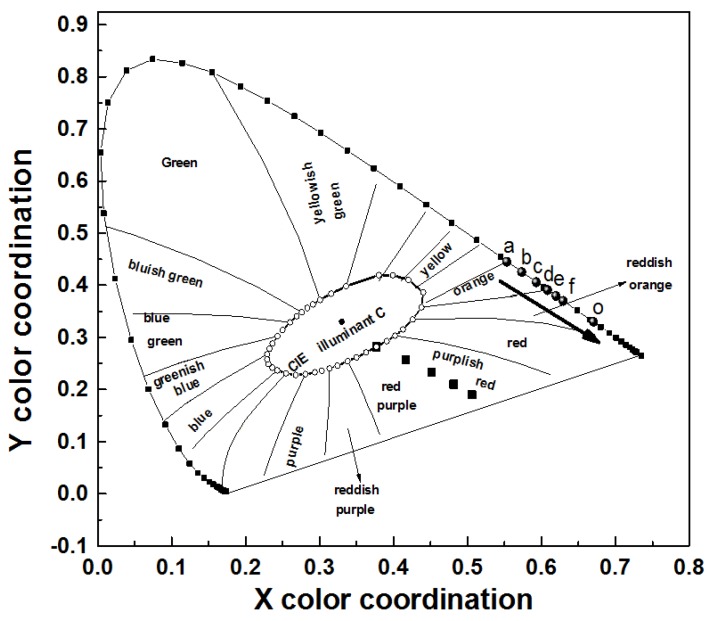
The chromaticity coordinates of LL_0.95−*y*_M:0.05Sm^3+^,*y*Eu^3+^ (*y* = 0 (a), 0.01 (b), 0.03 (c), 0.05 (d), 0.08 (e), 0.12 (f)) and the NTSC standard value point O.

LLM phosphors doped with Sm^3+^ ions emit orange-red light but the color coordinates are far from the standard red color coordinates of (0*.*670, 0*.*330), which limits applications in display products. In this work, the co-doping of Eu^3+^ has improved the color purity, so that the emission color is closer to that of standard red light. The color coordinates of the samples are shown in Figure 9 and, with the increase of the Eu^3+^ concentration, the sample points gradually approach to standard red light. The chromaticity coordinates of the LL_0.83_M:0.05Sm^3+^,0.12Eu^3+^ sample are (0.629, 0.370), which are close to the color coordinates (0.631, 0.350) of the commercial red phosphor Y_2_O_2_S:Eu^3+^. Hence, the luminous color of LL_0.95−*y*_M:0.05Sm^3+^,*y*Eu^3+^ can be adjusted so that it has application as a LED red phosphor.

### 3.5. Thermal Stability Analysis

**Figure 10:**
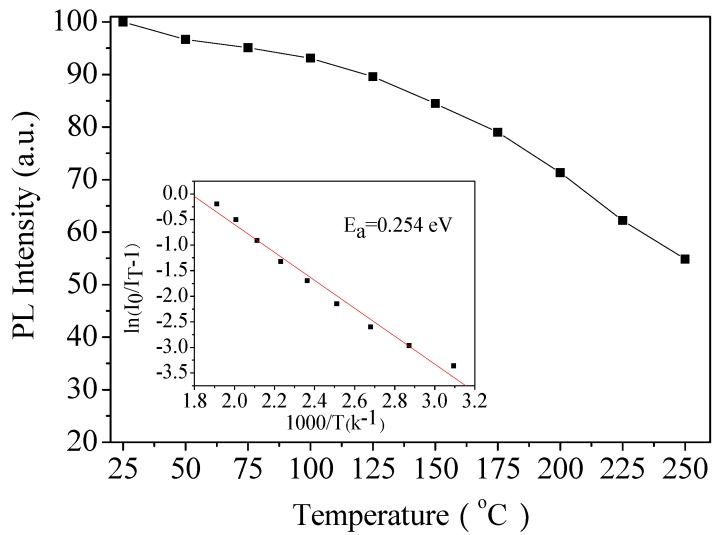
Temperature dependence of the PL intensity of LL_0.83_M:0.05Sm^3+^,0.12Eu^3+^ phosphor. Inset shows the fitting of the emission intensity according to the Arrhenius equation and the calculated activation energy are indicated in the inset.

The thermal quenching property of phosphor is an important parameter to be considered for application in high power LEDs because the junction temperature of typical LEDs can be above 100 °C. The thermal quenching of LL_0.83_M:0.05Sm^3+^,0.12Eu^3+^ phosphors was evaluated by measuring the temperature-dependent emission intensity from 25 to 250 °C. As shown in Figure 10, the temperature dependence of PL intensity indicated that the integrated emission intensity of the Eu^3+^ at 150 °C remained more than 85% of this measured at room temperature. In the inset (Figure 10) is shown the fitting of the emission intensity according to the following Arrhenius Equation (4) [30]:

(4) $$I_{T}=\frac{I_{0}}{1+c\exp(-\frac{E_{a}}{kT})}$$

where *I*_0_ and *I_T_* are the emission intensities at room and testing temperatures, respectively, *c* is a constant, and *k* is the Boltzmann constant ($k=8.617\times 10^{-5}\text{ eV}{\text{K}}^{-1}$). The corresponding activation energy (*Ea*) of thermal quenching is calculated to be 0.254 eV from the linear fitting slope of the $\ln(I_{0}/I_{T}-1)$ vs. 1000/*T* plot. The calculated activation energy of our sample is superior to that of Y_2_O_3_:Eu^3+^ commercial red phosphor [56], indicating that LL_0.83_M:0.05Sm^3+^,0.12Eu^3+^ has good enough thermal stability for LED application.

### 3.6. The Luminous Efficiency and the Performance for LED Applications

**Figure 11:**
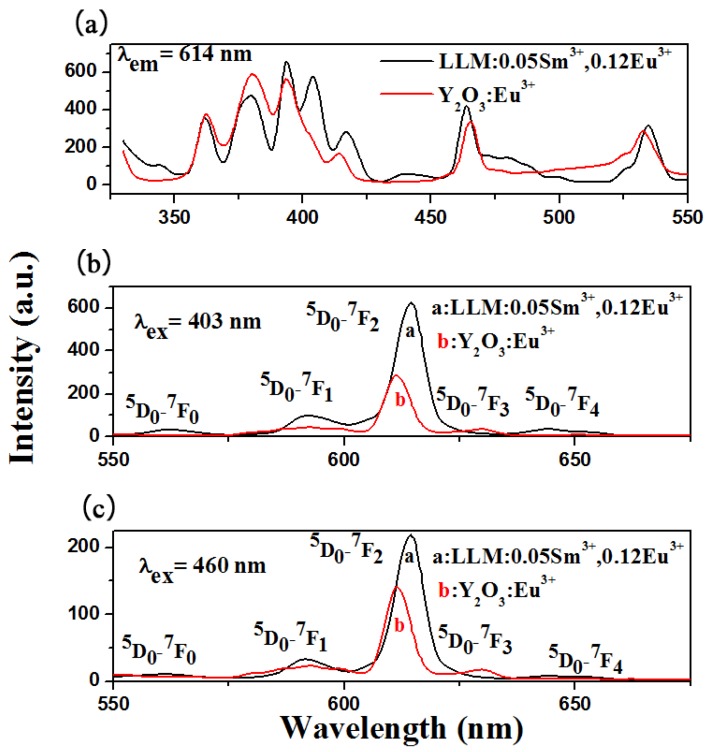
(**a**) The excitation spectra (λem = 614 nm); the emission spectra (**b**) (λex = 403 nm) and (**c**) (λex = 460 nm) of LL_0.83_M:0.05Sm^3+^,0.12Eu^3+^ and Y_2_O_3_:Eu^3+^.

The luminous efficiency of phosphor is an important parameter for practical application. The comparative emission spectra and excitation spectra between the LL_0.83_M:0.05Sm^3+^,0.12Eu^3+^ phosphor and commercial Y_2_O_3_:0.06Eu^3+^ red phosphor measured under the same experiment conditions are shown in Figure 11. Under 403 or 460 nm excitation, the LL_0.83_M:0.05Sm^3+^,0.12Eu^3+^ phosphor exhibits stronger emission than the commercial red Y_2_O_3_:Eu^3+^ phosphor.

**Figure 12:**
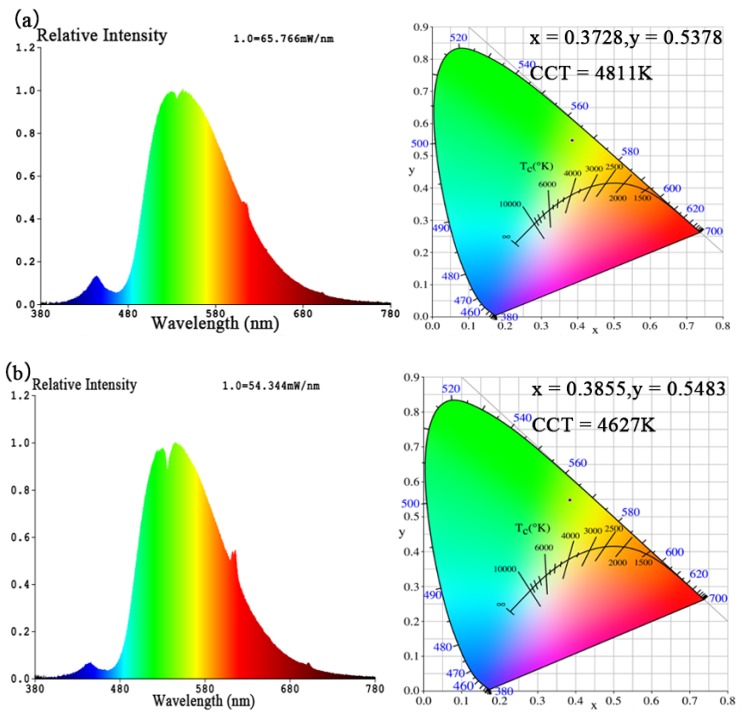

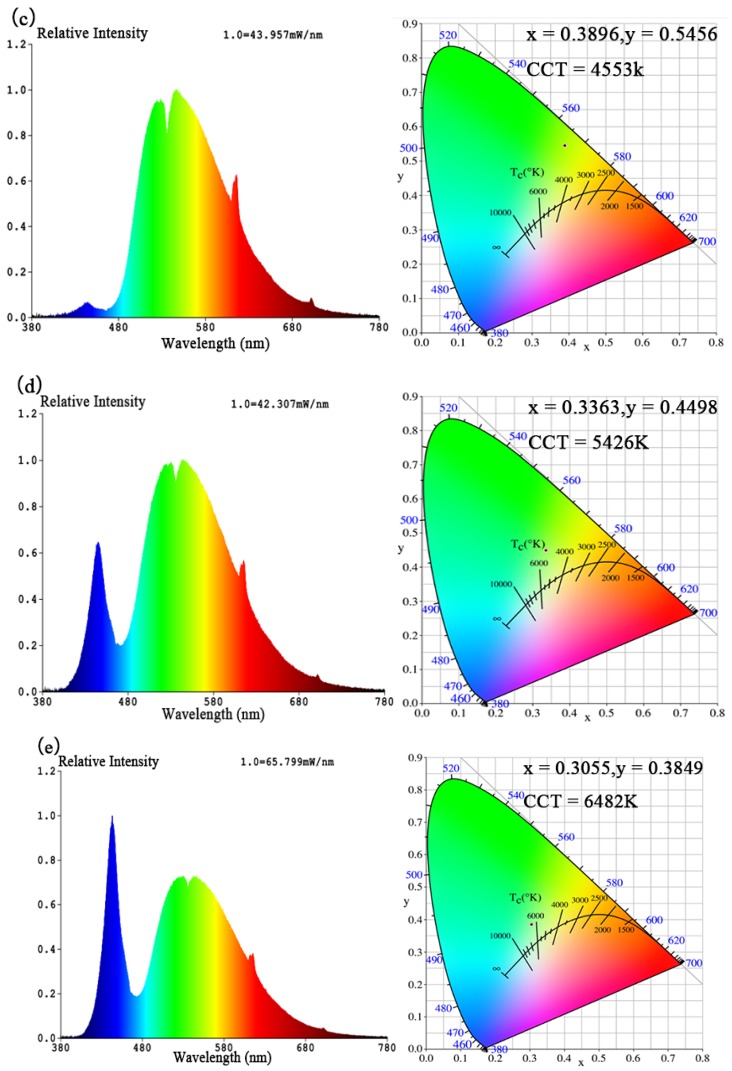
The emission spectra and CIE diagrams of the WLED lamps coated with LL_0.83_M:0.05Sm^3+^,0.12Eu^3+^ red phosphors and commercial yellow phosphors of different mass ratios, the mass ratios of phosphor glues coating on the chip of WLED lamps as shown in (**a**–**f**) are written in Table 1.

To estimate the potential of the Sm^3+^/Eu^3+^ co-doped LiLa(MoO_4_)_2_ phosphor for application, WLED lamps were fabricated by coating 455 nm blue LED chips with LL_0.83_M:0.05Sm^3+^,0.12Eu^3+^ red phosphor and commercial yellow phosphor of different mass ratios. The emission spectra and the CIE diagrams of the obtained WLED lamps are shown in Figure 12, while the color and electrical parameters are summarized in Table 1. The emission spectra of the WLED lamps exhibited a blue band at 455 nm which emits from the blue chip, a yellow band at 537 nm which emits from the yellow phosphors and a red peak at 615.7 nm which comes from our samples.

In Figure 12a–c, with the mass ratio of red phosphors increasing from 0.2 to 0.8, while that of yellow phosphor fixed at 0.2, the CCT is decreased from 4811 K to 4553 K and the color purity is increased from 73.6% to 81.0%, which indicate that the addition of the LL_0.83_M:0.05Sm^3+^,0.12Eu^3+^ red phosphors can improve the color purity and reduce the CCT of WLED lamps. However, for the cases, the CRI is low, which can be improved by decreasing the mass of yellow phosphors. The emission spectrum and the CIE diagram of the WLED lamp with red phosphor mass ratio of 0.52 and yellow phosphor mass ratio decreasing to 0.1, are given in Figure 12d. As can be found from Table 1, the CRI, chromaticity coordinates, CCT, luminous efficacy and color purity are 62.6, (0.3363, 0.4498), 5426 K and 107.08 lm W^−1^, 36.2% respectively, and the CRI is indeed improved. From Figure 12d, it can be noticed that the blue band is comparatively weak, which should be improved by decreasing the absorption of blue light, so as to obtain white light emission. For the purpose, the phosphor glue coating on the chip was thinned, and the emission spectrum and the CIE diagram of the WLED lamp are shown in Figure 12e. Now, the CIE indeed approaches the white light region, and the CRI, chromaticity coordinates, CCT, luminous efficacy and color purity are 65.0, (0.3055, 0.3849), 6482 K and 121.36 lm W^−1^, 10.4% respectively. The performance of the WLED lamps can be further optimized by adjusting the particle size and shape, the particle size distribution of the phosphor and the ratio of yellow and red phosphor. The results indicate that the phosphor-coated white LED had a high luminous efficacy and low CCT, demonstrating that the LL_0.83_M:0.05Sm^3+^,0.12Eu^3+^ is a very promising WLED red phosphor.

## 4. Conclusions

The Sm^3+^/Eu^3+^ co-doped LiLa(MoO_4_)_2_ phosphor was prepared by the traditional solid state method of sintering the reactants for 6 h in an ambient air atmosphere at 610 °C. The excitation spectra show that LL_1−*x*_M:*x*Sm^3+^ can effectively absorb photons of 403 and 460 nm wavelengths and this absorption matches well with the near UV LED and blue chip emission. The optimal dopant concentration of Sm^3+^ is *x* = 0.05, as evident from the emission spectra of LL_1−*x*_M:*x*Sm^3+^, and higher concentrations exhibit concentration quenching by the electric dipole—electric dipole mechanism.

With Sm^3+^ co-doping, the absorption spectrum of LLM:Eu^3+^ in the near ultraviolet and blue region becomes wider and stronger. Due to the energy transfer from Sm^3+^ to Eu^3+^, the Eu^3+^ emission in the co-doped phosphor can be effectively excited by 403 nm and 460 nm photons. The Sm^3+^ ions play the significant role in regulating luminous efficiency and color coordinates of the co-doped phosphor. The color coordinates of LL_0.83_M:0.05Sm^3+^,0.12Eu^3+^ are (0.629, 0.370) under 403 nm excitation and they are close to these of the standard red light point O (0.670, 0.330).

The LL_0.83_M:0.05Sm^3+^,0.12Eu^3+^ phosphor exhibits stronger emission than the commercial red Y_2_O_3_:Eu^3+^ phosphor under both 403 and 460 nm excitations, and it has superior thermal stability in comparison with that of the commercial red Y_2_O_3_:Eu^3+^ phosphor. The red LL_0.83_M:0.05Sm^3+^,0.12Eu^3+^ phosphor-precoated WLED lamp has a high luminous efficacy and a low CCT. In summary, LL_1−*x*−*y*_M:*x*Sm^3+^,*y*Eu^3+^ is a very promising WLED red phosphor.

## Figures and Tables

**Table 1:** The color and electrical parameters of WLEDs coated with phosphor glues of different mass ratios.

Figure 12	ENA6550 A:B:Yellow Phosphor:LLM(Eu,Sm)	CCT (K)	Color Purity (%)	CRI	CIE (*x*,* y*)		Luminous Efficacy (lm/w)	Voltage (V)	Current (A)	Power (W)
					*x*	*y*				
(a)	1.2:0.4:0.2:0.2	4811	73.6	50.1	0.3728	0.5378	158.15	37.6	0.6	22.6
(b)	1.2:0.4:0.2:0.5	4627	80.6	45.6	0.3855	0.5483	133.43	36.8	0.6	22.1
(c)	1.2:0.4:0.2:0.8	4553	81.0	46.4	0.3896	0.5456	107.20	36.8	0.6	22.1
(d)	1.2:0.4:0.1:0.52	5426	36.2	62.6	0.3363	0.4498	107.08	36.6	0.6	22.0
(e)	1.2:0.4:0.1:0.52	6482	10.4	65.0	0.3055	0.3849	121.36	36.8	0.6	22.1
